# (*E*)-1-(4-Meth­oxy­benzyl­idene)-2-phenyl­hydrazine

**DOI:** 10.1107/S160053681002533X

**Published:** 2010-07-03

**Authors:** Muhammad Mufakkar, M. Nawaz Tahir, Muhammad Ilyas Tariq, Shahbaz Ahmad, Muhammad Sarfraz

**Affiliations:** a Department of Chemistry, Government College University, Lahore, Pakistan; b Department of Physics, University of Sargodha, Sargodha, Pakistan; c Department of Chemistry, University of Sargodha, Sargodha, Pakistan

## Abstract

In the title compound, C_14_H_14_N_2_O, the dihedral angle between the aromatic rings is 9.30 (6)°. In the crystal, mol­ecules are linked by C—H⋯π and N—H⋯π inter­actions.

## Related literature

For related structures, see: Tunç *et al.* (2003 ▶); Harada *et al.* (2004 ▶).
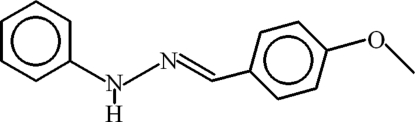


## Experimental

### 

#### Crystal data

C_14_H_14_N_2_O
*M* *_r_* = 226.27
Monoclinic, 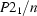

*a* = 5.8021 (2) Å
*b* = 7.5819 (2) Å
*c* = 27.7907 (9) Å
β = 95.808 (1)°
*V* = 1216.26 (7) Å^3^
*Z* = 4
Mo *K*α radiation
μ = 0.08 mm^−1^
*T* = 296 K
0.30 × 0.16 × 0.14 mm

#### Data collection

Bruker Kappa APEXII CCD diffractometer
Absorption correction: multi-scan (*SADABS*; Bruker, 2005 ▶) *T*_min_ = 0.942, *T*_max_ = 0.959
18675 measured reflections
3004 independent reflections
2257 reflections with *I* > 2σ(*I*)
*R*_int_ = 0.028

#### Refinement

*R*[*F* ^2^ > 2σ(*F* ^2^)] = 0.045
*wR*(*F* ^2^) = 0.124
*S* = 1.01
3004 reflections
155 parameters
H-atom parameters constrained
Δρ_max_ = 0.17 e Å^−3^
Δρ_min_ = −0.16 e Å^−3^

### 

Data collection: *APEX2* (Bruker, 2009 ▶); cell refinement: *SAINT* (Bruker, 2009 ▶); data reduction: *SAINT*; program(s) used to solve structure: *SHELXS97* (Sheldrick, 2008 ▶); program(s) used to refine structure: *SHELXL97* (Sheldrick, 2008 ▶); molecular graphics: *ORTEP-3 for Windows* (Farrugia, 1997 ▶) and *PLATON* (Spek, 2009 ▶); software used to prepare material for publication: *WinGX* (Farrugia, 1999 ▶) and *PLATON*.

## Supplementary Material

Crystal structure: contains datablocks global, I. DOI: 10.1107/S160053681002533X/hb5529sup1.cif

Structure factors: contains datablocks I. DOI: 10.1107/S160053681002533X/hb5529Isup2.hkl

Additional supplementary materials:  crystallographic information; 3D view; checkCIF report

## Figures and Tables

**Table 1:** Hydrogen-bond geometry (Å, °) *Cg*1 is the centroid of the C8–C13 phenyl ring.

*D*—H⋯*A*	*D*—H	H⋯*A*	*D*⋯*A*	*D*—H⋯*A*
N1—H1⋯*Cg*1^i^	0.86	2.69	3.3484 (13)	146
C3—H3⋯*Cg*1^ii^	0.93	2.63	3.3796 (14)	138

Symmetry codes: (i) 
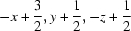
; (ii) 
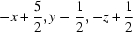
.

**Fig. 1.**
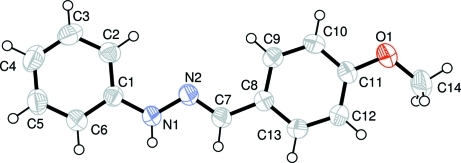
View of (I) with displacement ellipsoids drawn at the 50% probability level. H-atoms are shown by circles of arbitrary radius.

## supplementary crystallographic information

### Comment

The crystal structure of (II) *i.e.*, *N*-(4-methoxybenzylidene)-N^'^-(2-pyridyl)hydrazine (Tunç *et al.* 2003) and *N*-(4-methoxybenzylidene)aniline (Harada *et al.* 2004) have been published which are related to the title compound (I, Fig. 1).

In (I) the phenyl ring A (C1–C6) of phenylhydrazide and B (C8–C13) of 4-anisaldehyde are planar with r. m. s. deviation of 0.0015 and 0.0096 Å, respectively. The dihedral angle between A/B is 9.30 (6)°. The central group C (N1/N2/C7) is of course planar and the orientation of A/C and B/C is 11.59 (17) and 2.89 (18)°, respectively. The molecules are essentially monomer. Due to the packing and unavailabilty of strong acceptor atom, the H-atom of N—H is not directly involved in H-bonding. The molecules are stabilized through C—H···π and N—H···π interactions (Table 1).

### Experimental

Equimolar quantities of phenylhydrazine and 4-methoxybenzaldehyde were refluxed in methanol for 45 min resulting in yellow solution. The solution was kept at room temperature which affoarded yellow needles of (I) after 72 h.

### Refinement

Although all H-atoms appear in the difference Fourier map but were positioned geometrically (N–H = 0.86, C–H = 0.93–0.96 Å) and refined as riding with *U*_iso_(H) = *xU*_eq_(C, N), where *x* = 1.5 for methyl and *x* = 1.2 for all other H-atoms.

### Crystal data

**Table:**

C_14_H_14_N_2_O	*F*(000) = 480
*M**_r_* = 226.27	*D*_x_ = 1.236 Mg m^−^^3^
Monoclinic, *P*2_1_/*n*	Mo *K*α radiation, λ = 0.71073 Å
Hall symbol: -P 2yn	Cell parameters from 2257 reflections
*a* = 5.8021 (2) Å	θ = 2.8–28.4°
*b* = 7.5819 (2) Å	µ = 0.08 mm^−^^1^
*c* = 27.7907 (9) Å	*T* = 296 K
β = 95.808 (1)°	Cut needle, yellow
*V* = 1216.26 (7) Å^3^	0.30 × 0.16 × 0.14 mm
*Z* = 4	

### Data collection

**Table:**

Bruker Kappa APEXII CCD diffractometer	3004 independent reflections
Radiation source: fine-focus sealed tube	2257 reflections with *I* > 2σ(*I*)
graphite	*R*_int_ = 0.028
Detector resolution: 8.20 pixels mm^-1^	θ_max_ = 28.4°, θ_min_ = 2.8°
ω scans	*h* = −7→7
Absorption correction: multi-scan (*SADABS*; Bruker, 2005)	*k* = −9→10
*T*_min_ = 0.942, *T*_max_ = 0.959	*l* = −37→37
18675 measured reflections	

### Refinement

**Table:**

Refinement on *F*^2^	Primary atom site location: structure-invariant direct methods
Least-squares matrix: full	Secondary atom site location: difference Fourier map
*R*[*F*^2^ > 2σ(*F*^2^)] = 0.045	Hydrogen site location: inferred from neighbouring sites
*wR*(*F*^2^) = 0.124	H-atom parameters constrained
*S* = 1.01	*w* = 1/[σ^2^(*F*_o_^2^) + (0.0582*P*)^2^ + 0.2017*P*] where *P* = (*F*_o_^2^ + 2*F*_c_^2^)/3
3004 reflections	(Δ/σ)_max_ < 0.001
155 parameters	Δρ_max_ = 0.17 e Å^−^^3^
0 restraints	Δρ_min_ = −0.16 e Å^−^^3^

### Special details

**Table:**

Geometry. Bond distances, angles *etc*. have been calculated using the rounded fractional coordinates. All su's are estimated from the variances of the (full) variance-covariance matrix. The cell e.s.d.'s are taken into account in the estimation of distances, angles and torsion angles
Refinement. Refinement of *F*^2^ against ALL reflections. The weighted *R*-factor *wR* and goodness of fit *S* are based on *F*^2^, conventional *R*-factors *R* are based on *F*, with *F* set to zero for negative *F*^2^. The threshold expression of *F*^2^ > σ(*F*^2^) is used only for calculating *R*-factors(gt) *etc*. and is not relevant to the choice of reflections for refinement. *R*-factors based on *F*^2^ are statistically about twice as large as those based on *F*, and *R*- factors based on ALL data will be even larger.

### Fractional atomic coordinates and isotropic or equivalent isotropic displacement parameters (Å^2^)

**Table:**

	*x*	*y*	*z*	*U*_iso_*/*U*_eq_	
O1	0.58571 (17)	0.12817 (13)	0.05523 (3)	0.0593 (3)	
N1	1.0199 (2)	0.27807 (16)	0.32418 (4)	0.0576 (4)	
N2	0.99619 (18)	0.22429 (14)	0.27710 (4)	0.0475 (3)	
C1	1.1991 (2)	0.21880 (15)	0.35653 (4)	0.0431 (4)	
C2	1.3896 (2)	0.13069 (16)	0.34188 (5)	0.0486 (4)	
C3	1.5625 (2)	0.07433 (18)	0.37608 (6)	0.0594 (5)	
C4	1.5495 (3)	0.1038 (2)	0.42465 (6)	0.0659 (5)	
C5	1.3613 (3)	0.1917 (2)	0.43908 (5)	0.0616 (5)	
C6	1.1872 (2)	0.24896 (17)	0.40553 (5)	0.0520 (4)	
C7	0.8064 (2)	0.26802 (16)	0.25267 (4)	0.0458 (4)	
C8	0.7500 (2)	0.22464 (14)	0.20197 (4)	0.0404 (3)	
C9	0.9001 (2)	0.13387 (15)	0.17420 (4)	0.0436 (4)	
C10	0.8384 (2)	0.10202 (16)	0.12601 (4)	0.0458 (4)	
C11	0.6270 (2)	0.16101 (15)	0.10379 (4)	0.0438 (4)	
C12	0.4738 (2)	0.24744 (16)	0.13072 (4)	0.0458 (4)	
C13	0.5363 (2)	0.27705 (16)	0.17934 (4)	0.0449 (4)	
C14	0.3755 (3)	0.1915 (3)	0.03100 (5)	0.0771 (6)	
H1	0.92065	0.35071	0.33393	0.0691*	
H2	1.40053	0.10982	0.30922	0.0583*	
H3	1.69010	0.01538	0.36620	0.0713*	
H4	1.66685	0.06457	0.44740	0.0791*	
H5	1.35170	0.21259	0.47179	0.0739*	
H6	1.06052	0.30834	0.41570	0.0624*	
H7	0.69829	0.33119	0.26824	0.0550*	
H9	1.04277	0.09490	0.18855	0.0523*	
H10	0.93876	0.04044	0.10807	0.0550*	
H12	0.33060	0.28509	0.11628	0.0550*	
H13	0.43235	0.33379	0.19749	0.0539*	
H14A	0.24713	0.13841	0.04481	0.1156*	
H14B	0.36847	0.16173	−0.00269	0.1156*	
H14C	0.36828	0.31731	0.03446	0.1156*	

### Atomic displacement parameters (Å^2^)

**Table:**

	*U*^11^	*U*^22^	*U*^33^	*U*^12^	*U*^13^	*U*^23^
O1	0.0679 (6)	0.0706 (6)	0.0389 (5)	0.0039 (5)	0.0023 (4)	−0.0054 (4)
N1	0.0616 (7)	0.0695 (7)	0.0400 (6)	0.0237 (6)	−0.0038 (5)	−0.0104 (5)
N2	0.0537 (6)	0.0484 (6)	0.0398 (6)	0.0048 (4)	0.0018 (5)	−0.0027 (4)
C1	0.0458 (6)	0.0395 (6)	0.0431 (7)	0.0005 (5)	0.0004 (5)	−0.0025 (5)
C2	0.0480 (7)	0.0464 (6)	0.0512 (7)	−0.0004 (5)	0.0045 (5)	−0.0080 (5)
C3	0.0476 (7)	0.0518 (7)	0.0771 (10)	0.0065 (6)	−0.0020 (7)	−0.0102 (7)
C4	0.0627 (9)	0.0603 (8)	0.0690 (10)	0.0073 (7)	−0.0207 (7)	−0.0022 (7)
C5	0.0714 (9)	0.0650 (9)	0.0458 (8)	0.0017 (7)	−0.0066 (7)	−0.0033 (6)
C6	0.0560 (7)	0.0546 (8)	0.0452 (7)	0.0065 (6)	0.0035 (6)	−0.0055 (5)
C7	0.0503 (7)	0.0449 (6)	0.0421 (7)	0.0051 (5)	0.0038 (5)	−0.0012 (5)
C8	0.0444 (6)	0.0370 (5)	0.0400 (6)	−0.0027 (4)	0.0048 (5)	0.0016 (4)
C9	0.0412 (6)	0.0434 (6)	0.0462 (7)	0.0004 (5)	0.0044 (5)	0.0036 (5)
C10	0.0465 (6)	0.0467 (6)	0.0458 (7)	0.0008 (5)	0.0125 (5)	−0.0029 (5)
C11	0.0509 (7)	0.0430 (6)	0.0376 (6)	−0.0061 (5)	0.0050 (5)	−0.0006 (5)
C12	0.0413 (6)	0.0495 (7)	0.0457 (7)	−0.0009 (5)	−0.0004 (5)	−0.0010 (5)
C13	0.0435 (6)	0.0460 (6)	0.0456 (7)	0.0027 (5)	0.0070 (5)	−0.0024 (5)
C14	0.0929 (12)	0.0906 (12)	0.0443 (8)	0.0169 (10)	−0.0095 (8)	−0.0024 (8)

### Geometric parameters (Å, °)

**Table:**

O1—C11	1.3694 (14)	C10—C11	1.3902 (16)
O1—C14	1.416 (2)	C11—C12	1.3836 (16)
N1—N2	1.3641 (16)	C12—C13	1.3815 (16)
N1—C1	1.3792 (16)	C2—H2	0.9300
N2—C7	1.2776 (16)	C3—H3	0.9300
N1—H1	0.8600	C4—H4	0.9300
C1—C6	1.3894 (18)	C5—H5	0.9300
C1—C2	1.3874 (17)	C6—H6	0.9300
C2—C3	1.3782 (19)	C7—H7	0.9300
C3—C4	1.378 (2)	C9—H9	0.9300
C4—C5	1.373 (2)	C10—H10	0.9300
C5—C6	1.374 (2)	C12—H12	0.9300
C7—C8	1.4516 (16)	C13—H13	0.9300
C8—C13	1.3903 (16)	C14—H14A	0.9600
C8—C9	1.4013 (16)	C14—H14B	0.9600
C9—C10	1.3722 (16)	C14—H14C	0.9600
			
C11—O1—C14	117.60 (10)	C3—C2—H2	120.00
N2—N1—C1	121.61 (11)	C2—C3—H3	119.00
N1—N2—C7	115.47 (11)	C4—C3—H3	119.00
N2—N1—H1	119.00	C3—C4—H4	120.00
C1—N1—H1	119.00	C5—C4—H4	120.00
N1—C1—C2	122.42 (11)	C4—C5—H5	120.00
N1—C1—C6	118.45 (11)	C6—C5—H5	120.00
C2—C1—C6	119.14 (11)	C1—C6—H6	120.00
C1—C2—C3	119.53 (12)	C5—C6—H6	120.00
C2—C3—C4	121.14 (13)	N2—C7—H7	118.00
C3—C4—C5	119.26 (14)	C8—C7—H7	118.00
C4—C5—C6	120.46 (13)	C8—C9—H9	120.00
C1—C6—C5	120.47 (12)	C10—C9—H9	120.00
N2—C7—C8	123.68 (11)	C9—C10—H10	120.00
C9—C8—C13	117.82 (10)	C11—C10—H10	120.00
C7—C8—C9	123.66 (10)	C11—C12—H12	120.00
C7—C8—C13	118.52 (10)	C13—C12—H12	120.00
C8—C9—C10	120.57 (11)	C8—C13—H13	119.00
C9—C10—C11	120.55 (11)	C12—C13—H13	119.00
O1—C11—C12	124.15 (11)	O1—C14—H14A	109.00
O1—C11—C10	115.96 (10)	O1—C14—H14B	109.00
C10—C11—C12	119.89 (10)	O1—C14—H14C	109.00
C11—C12—C13	119.12 (11)	H14A—C14—H14B	109.00
C8—C13—C12	121.99 (11)	H14A—C14—H14C	109.00
C1—C2—H2	120.00	H14B—C14—H14C	109.00
			
C14—O1—C11—C12	1.90 (19)	C4—C5—C6—C1	0.0 (2)
C14—O1—C11—C10	−177.93 (13)	N2—C7—C8—C13	179.04 (12)
N2—N1—C1—C2	13.07 (18)	N2—C7—C8—C9	−1.57 (19)
N2—N1—C1—C6	−166.94 (11)	C7—C8—C13—C12	177.18 (11)
C1—N1—N2—C7	170.35 (11)	C9—C8—C13—C12	−2.24 (17)
N1—N2—C7—C8	179.36 (11)	C7—C8—C9—C10	−177.96 (11)
N1—C1—C6—C5	179.76 (12)	C13—C8—C9—C10	1.44 (17)
N1—C1—C2—C3	−179.78 (12)	C8—C9—C10—C11	0.73 (18)
C6—C1—C2—C3	0.24 (18)	C9—C10—C11—C12	−2.17 (18)
C2—C1—C6—C5	−0.26 (19)	C9—C10—C11—O1	177.67 (11)
C1—C2—C3—C4	0.1 (2)	O1—C11—C12—C13	−178.45 (11)
C2—C3—C4—C5	−0.3 (2)	C10—C11—C12—C13	1.38 (18)
C3—C4—C5—C6	0.3 (2)	C11—C12—C13—C8	0.85 (18)

### Hydrogen-bond geometry (Å, °)

**Table:**

Cg1 is the centroid of the C8–C13 phenyl ring.

**Table:**

*D*—H···*A*	*D*—H	H···*A*	*D*···*A*	*D*—H···*A*
N1—H1···Cg1^i^	0.86	2.69	3.3484 (13)	146
C3—H3···Cg1^ii^	0.93	2.63	3.3796 (14)	138

Symmetry codes: (i) −*x*+3/2, *y*+1/2, −*z*+1/2; (ii) −*x*+5/2, *y*−1/2, −*z*+1/2.
